# Optimal Vitamin D Supplementation Levels for Cardiovascular Disease Protection

**DOI:** 10.1155/2015/864370

**Published:** 2015-09-08

**Authors:** Sebastian T. Lugg, Phillip A. Howells, David R. Thickett

**Affiliations:** School of Clinical and Experimental Medicine, College of Medical and Dental Sciences, Centre for Translational Inflammation Research (CTIR), University of Birmingham Laboratories, Queen Elizabeth Hospital Birmingham, Birmingham B15 2TH, UK

## Abstract

First described in relation to musculoskeletal disease, there is accumulating data to suggest that vitamin D may play an important role in cardiovascular disease (CVD). In this review we aim to provide an overview of the role of vitamin D status as both a marker of and potentially causative agent of hypertension, coronary artery disease, heart failure, atrial fibrillation, stroke, and peripheral vascular disease. The role of vitamin D levels as a disease marker for all-cause mortality is also discussed. We review the current knowledge gathered from experimental studies, observational studies, randomised controlled trials, and subsequent systematic reviews in order to suggest the optimal vitamin D level for CVD protection.

## 1. Vitamin D Introduction

**Figure 1:**
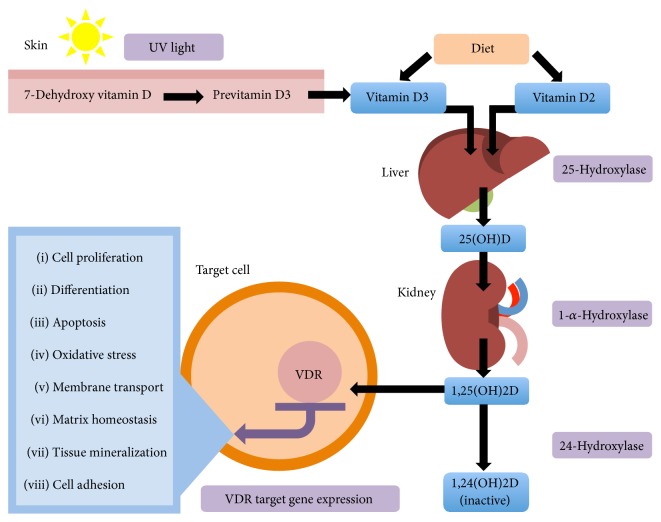
Vitamin D metabolism and the vitamin D receptor.

Vitamin D is a fat-soluble vitamin that functions as a steroid hormone. In the skin, ultraviolet (UV) light causes photochemical cleavage of 7-dehydrocholesterol into previtamin D3, which spontaneously isomerises to form vitamin D3 (cholecalciferol) [1] (vitamin D metabolism is demonstrated in Figure 1). Vitamin D2 (ergocalciferol) is a plant-derived form of vitamin D through exposure of yeast to UV light. Skin synthesis of vitamin D3 accounts for about 80% of vitamin D [2]; dietary sources include fish oils, egg yolks, mushrooms, dairy products, and fortified cereals.

## 2. Vitamin D Metabolism and Physiology

The inactive vitamin D precursors undergo first 25-hydroxylation in the liver to form 25-hydroxyvitamin D [25(OH)D]. This is the main circulating form of vitamin D and therefore is usually considered as a circulating biomarker for vitamin D status [3]. The vast majority (>99.95%) of 25(OH)D is bound to serum proteins and has a half-life of 2 to 3 weeks. The second step is further hydroxylation by 1-*α*-hydroxylase in the kidney resulting in 1,25-dihydroxyvitamin D [1,25(OH)2D] or calcitriol. 1-*α*-hydroxylase activity is modulated by parathyroid hormone (PTH) and fibroblast growth factor 23. Various other tissues also express 1-*α*-hydroxylase (e.g., endothelial cells, cardiomyocytes, and macrophages). Furthermore, all target cells express 24-hydroxylase that converts calcitriol into an inactive form 1,24(OH)2D. Because of calcitriol's short circulating half-life of only a few hours, it is a less suitable vitamin D status biomarker.

Most biological effects of vitamin D are likely to be mediated by calcitriol, whether produced locally in tissues or diffusing from the blood. The vitamin D receptor (VDR) has been found in many target tissues includes all the major cardiovascular cell types: endothelial cells [4], vascular smooth muscle (VSM) cells [5], cardiomyocytes [6], platelets [7], and most immune cells [8]. Gene expression is dependent on tissue specific coactivators and cosuppressors [9]. It is estimated that the VDR activation may regulate about 5% of the total genome [10], pathway analysis suggesting effects upon regulation of cell proliferation, differentiation, apoptosis, oxidative stress, membrane transport, matrix homeostasis, tissue mineralization, and cell adhesion [11].

## 3. Vitamin D Deficiency

A key role of calcitriol is to increase the intestinal absorption of both calcium and phosphorus. The hallmark of vitamin D deficiency is elevated PTH levels; a hormone stimulated as a result of low serum calcium levels. PTH causes increased intestinal and renal calcitriol production, calcium to be extruded from bone, and renal calcium retention. PTH is known to have direct adverse cardiac effects, including endothelial dysfunction, hypertension, increased aortic stiffness, dyslipidemia, hyperglycemia, and subclinical aortic valve calcification. Furthermore, elevated PTH is associated with increased cardiac deaths [12]. Classical clinical consequences of vitamin D deficiency include proximal myopathy and myalgia, which may in part explain the increased risk of functional limitations: falling and fractures in elderly patients who are vitamin D deficient [13–15].

Vitamin D's effect on calcium homeostasis and the level necessary to avoid a rise in PTH levels are commonly used for the definition of adequate vitamin D status, which is quoted as ≥100 nmol/L (40 ng/mL) [16]. Therefore, serum 25(OH)D levels between 100 and 150 nmol/L are ideal, levels below 50 nmol/L are associated with severe vitamin D deficiency, and levels between 50 and 74 nmol/L are described as moderate vitamin D deficiency or insufficiency [17] (Table 1). Vitamin D toxicity, however, would only be expected at levels >375 nmol/L [18]. A review for the UK Government suggested a safe upper limit with low risk of toxicity for most adults at approximately 25–50 micrograms/day (1000–2000 IU/d), with references to sources suggesting much higher doses may be acceptably safe [19].

Vitamin D insufficiency is prevalent in almost half of the healthy population of developed countries [17]. Risk factors for low 25(OH)D levels are advanced age, female sex, darker skin pigmentation, less sunlight exposure (due to latitude, clothing, or prolonged periods indoors), reduced intake of vitamin D through diet, and the winter season [20].

## 4. Vitamin D and Hypertension

There are many potential mechanisms for vitamin D deficiency to produce hypertension. Direct effects are mediated via the renin-angiotensin system (RAS) or endothelial or VSM function. Indirect effects may include increased occurrence of diabetes mellitus, atherosclerosis, vascular calcification, and changes in renal structure and function [21].

The NHANES III study looked at serum 25(OH)D levels in relation to CVD risk factors in approximately 13,000 US adults and found vitamin D status was inversely associated with blood pressure. The authors estimated that about 50% of the difference in prevalence of hypertension between African and Caucasian Americans was due to vitamin D deficiency in African Americans [22] (key studies of vitamin D and CVD are summarised in Table 2). Other studies found 4 mmHg difference in systolic blood pressure between quartiles [23]. Blood pressure rises and falls with seasonal variation of sunlight, and 25(OH)D levels are inversely related to this [24]. Furthermore, blood pressure rises by 2.5 mmHg for each 10 degrees north or south of the equator [25], which may imply a dose dependent relationship.

A systematic review and meta-analysis investigated whether supplementation with vitamin D or its analogues reduce blood pressure; only randomised controlled trials (RCTs) comparing placebo to vitamin D supplementation for a minimum of 4 weeks were included [26]. This included 46 trials (4541 participants) in the trial-level meta-analysis. No effect of vitamin D supplementation was seen on systolic blood pressure (0.0 [95% CI, −0.8 to 0.8] mmHg; *P* = 0.97) or diastolic blood pressure (−0.1 [95% CI, −0.6 to 0.5] mmHg;  *P* = 0.84). Furthermore, similar results were found on the analysis of individual patient data obtained for 27 trials (3092 participants). The investigators found that subgroup analysis did not reveal any baseline factor predictive of a better response to therapy.

People of African-Caribbean ethnicity have significantly higher rates of hypertension than Caucasian people, which may be due to the higher skin content of melanin reducing vitamin D3 production. A recently published 4-arm placebo controlled RCT investigated the effect of supplementation of 1000, 2000, and 4000 IU of vitamin D3 per day on blood pressure in an unselected population of people of African-Caribbean ethnicity [27]. At 3 months, there was a statistically significant reduction of 1.4 mmHg in the systolic blood pressure of participants for each additional 1000 IU/d of vitamin D supplementation (*P* = 0.04) (−1.4 mmHg for placebo versus 4.0 mmHg for 4000 IU/d), whilst there was no significant reduction in the diastolic blood pressure (*P* = 0.37). Another study in African-Caribbean youths found supplementing 2000 IU/d may be effective at optimising vitamin D status and reducing aortic stiffness [28].

Overall, blood pressure changes in response to changes in serum 25(OH)D levels are detected inconsistently and are small when detected, thus not reaching clinical significance, although this may reflect the baseline characteristics, study duration, or differing dosage. Further research is needed in this field. Of interest will be the future data from the DO-HEALTH study [29], a large randomised clinical trial (*n* = 2158) sponsored by the European Union, which aims to establish whether 2000 IU/d vitamin D, omega-3 fatty acids, and a simple home exercise program will prevent disease at older age, including blood pressure among other parameters as a primary endpoint.

## 5. Vitamin D and Heart Failure

There is increasing evidence that vitamin D has modulatory effects on mechanisms known to be important in heart failure (HF). Animal models have shown that calcitriol has been shown to have a key role in enabling the maturation and differentiation of ventricular myocytes isolated from neonatal rat hearts [30] and VDR knockout mice exhibit increased ventricular mass and higher levels of atrial natriuretic peptide, which lead to ventricular dilatation and impaired electromechanical association [31–33].

In observational studies, the incidence of chronic HF has been shown to be greater in cohorts with low vitamin D status [34]. However, the ICELAND-MI study looking at an older-aged community cohort showed no association between serum 25(OH)D and magnetic resonance imaging measures of cardiac function, whereas elevated PTH was associated with lower ejection fraction and increased left ventricular mass [35]. Other studies have shown elevated PTH to be associated with HF [36], and left ventricular hypertrophy [37], possibly suggesting vitamin D deficiency, may be a marker of elevated PTH levels.

An RCT of 80 infants with chronic congestive HF investigated the effects of 12 weeks of vitamin D (1000 IU) versus placebo on cardiac function. The baseline 25(OH)D levels were low; 25(OH)D levels significantly rose in the intervention group and were associated with both improvement in cardiac function and a marked increase in interleukin- (IL-) 10 and decreased PTH, IL-6, and TNF-*α* [38]. In adults with HF, a RCT of placebo verses vitamin D supplementation for 9 months was associated with a 67 nmol/L rise in 25(OH)D levels; there were significantly reduced levels of proinflammatory cytokines TNF-*α*, IL-10, and PTH in the treatment group compared to placebo [39]. Vitamin D deficiency not only is more common in HF compared to non-HF controls 25(OH)D <25 nmol/L (28% versus 22%) but also has been shown to be an independent predictor for increased mortality in HF patients (hazard ratio [HR] 1.52; 95% CI, 1.21–1.92), and supplementation has been shown to offer survival benefit from a retrospective database analysis (HR, 0.68; 95% CI, 0.54–0.85; *P* < 0.0001) [40].

## 6. Vitamin D and Atrial Fibrillation

Vitamin D may play a role in pathogenesis of atrial fibrillation (AF). Proposed mechanisms include the indirect effect of vitamin D on the atria through RAS and modulation in levels of reactive oxygen species, which contribute to inflammation and proarrhythmic substrate formation [41]. Large observational studies find the incidence of AF to be higher in winter than in summer months [42, 43], correlating with seasonal variation in 25(OH)D levels. However, this does not imply causality, since the lower winter temperature and the higher incidence of respiratory tract infections are risk factors for AF. The Framingham Heart Study showed no association between 25(OH)D levels and AF in either original or offspring cohorts [44] and no association has been demonstrated between 25(OH)D levels and different types of AF (either paroxysmal, persistent, or permanent) [45]. Other studies have shown significantly lower 25(OH)D levels in patients with nonvalvular AF [46]; those with levels <50 nmol/L have a twofold higher incidence of nonvalvular AF than those with levels >75 nmol/L [47]. Another observational study showed vitamin D deficiency to be associated with new-onset AF in a hypertensive cohort [48].

## 7. Vitamin D and Coronary Artery Disease

Vitamin D exerts a variety of direct effects relating to atherosclerosis such as modulating endothelial function and influencing VSM proliferation and migration; indirect effects occur through dysglycemia, dyslipidemia, and RAS [49]. In the LURIC Study, a large cohort of subjects (*n* = 1801) referred for coronary angiography found 92% of individuals had suboptimal 25(OH)D levels (<75 nmol/L) and 22% were severely deficient (<25 nmol/L) [50]. Furthermore, after adjustment, those patients with optimal levels showed a substantial reduction in all-cause mortality (HR, 0.25; 95% CI, 0.13–0.46) and cardiovascular mortality compared to those with severe deficiency. The Framingham Offspring Study found that individuals with 25(OH)D <37.5 nmol/L had a hazard ratio of 1.62 for development of CVD compared to those with a level of ≥37 nmol/L [51].

An intriguing cross-sectional and observational study looked at the effect of 25(OH)D levels <75 nmol/L on epicardial coronary flow rate, subclinical atherosclerosis, and endothelial function. The study enrolled 222 patients who had undergone coronary angiography for suspected ischemic heart disease and were found to have normal or near-normal coronary arteries. The incidence of slow coronary flow rate (SCF) was significantly higher in the vitamin D deficient group (relative risk [RR], 3.5; 95% CI, 1.1–10.5; *P* = 0.01) and after adjusting for cardiovascular disease risk factors vitamin D insufficiency was an independent risk factor for SCF. In addition, vitamin D insufficiency was associated with endothelial dysfunction and subclinical atherosclerosis [52]. Furthermore, in a study of 375 patients undergoing coronary angiography to assess the severity of coronary artery disease (CAD), levels of 25(OH)D were significantly lower in patients with CAD than in those without (57 ± 1.73 versus 70.1 ± 2.46 nmol/L; *P* < 0.01) [53]. The authors found vitamin D to be the most significant predictor for CAD and 25(OH)D levels were significantly lower in triple vessel compared to single vessel disease. However, there was no correlation between 25(OH)D levels and arterial stiffness using pulse wave velocity and peripheral artery disease using ankle brachial pressure index.

In assessing supplementation of vitamin D, the RECORD trial (*n* = 5292) was a factorial RCT that compared the effects of vitamin D3 (800 IU/d), calcium, vitamin D plus calcium, and placebo in cardiovascular events. The study found that vitamin D might protect against heart failure (HR, 0.75; 95% CI, 0.58–0.97) but does not appear to protect against myocardial infarction (MI) and stroke. The authors conducted further a systematic review and meta-analysis including 21 studies (*n* = 13,033), which reached the same conclusion [54]. Another meta-analysis of 51 RCTs found that vitamin D supplementation did not have a significant impact on MI (RR, 1.02; 95% CI, 0.93–1.13; *P* = 0.64) [55]. There is currently insufficient evidence that vitamin D supplementation is associated with CAD risk reduction.

## 8. Vitamin D and Stroke

There have been a number of observational studies linking vitamin D status with stroke as well as plausible animal data and imaging studies of carotid atheroma. The VDR in the brain modulates neuronal activity as well as potentially influencing vascular effects [56]. Elevated PTH has been associated with stroke as well as adverse cardiovascular outcomes [57]. An observational study from Denmark, which also included a meta-analysis, showed stepwise increase in stroke incidence for decreasing 25(OH)D level quartiles [58].

In order to test the hypothesis that supplementation may be beneficial, a group of people with previous strokes were given 10,000 IU of vitamin D2 or placebo. At 8 weeks, endothelial function was shown to improve using brachial artery ultrasound following an occlusion test, although other markers of cardiovascular health were not. The small numbers (*n* = 58), age of the cohort, and modest increase in vitamin D levels may be factors in the limited positive findings. The authors recommend a more nuanced approach to vitamin D supplementation than blanket delivery [59].

The very large Women's Health Initiative study [60] showed no significant difference in stroke with treatment (calcium and 200 IU vitamin D3 twice daily) versus placebo. However, this trial was not powered for cardiovascular endpoints, the inclusion of calcium supplementation may have counteracted the benefits of vitamin D, and vitamin D dose may have been too small [57]. Another RCT demonstrated that supplementation of vitamin D3 (800 IU) with/without calcium versus placebo was shown not to alter outcome for stroke or cardiovascular mortality [61]. Interestingly, an RCT using 10,000 IU delivered every 4 months was conducted which showed lower fracture rates and a nonsignificant trend to reduced mortality and cardiovascular events [62]. It has been suggested that lack of PTH sampling in RCTs makes it more difficult to ascertain adequate dosing [57].

Overall, the association between low vitamin D and stroke is clear but its causality is less clear and there has been a lack of benefit of supplementation demonstrated from clinical trials to date.

## 9. Vitamin D and Peripheral Vascular Disease

Observational studies suggest a role of vitamin D status in peripheral vascular disease (PVD); a retrospective database study from the US showed a 50% increase in PVD in the lowest tertile of 25(OH)D levels (<37.5 nmol/L) compared to the higher two tertiles [63]. Similarly, the data from the NHANES Study showed that after adjusting for risk factors those patients in the lowest quartile (<44.5 nmol/L) were 1.80 (CI 1.19–2.74) times more likely to have PVD compared to the highest quartile (>73 nmol/L) [64]. The study found no association between PVD and calcium, phosphate, and PTH. An older study demonstrated worsening PVD was associated with lower 25(OH)D levels but suggested that it could be that limited exercise and sunlight exposure from reduced mobility secondary to PVD that led to hypovitaminosis D rather than vitamin D deficiency being the cause or exacerbating factor in PVD [65].

Vitamin D may act to promote PVD by exacerbating traditional cardiovascular risk factors via mechanisms described above or by promoting myopathy, in turn limiting exercise and/or contributing to coexisting symptoms [66]. Alternatively, vitamin D may play a role in the pathobiology of atherosclerosis as in other vascular beds [67].

A detailed investigation of the elderly and vitamin D showed that those with PVD and low 25(OH)D (<75 nmol/L) had a faster decline in their 6-minute walk test over follow-up and also a faster decline in their Short Physical Performance Battery (a score for assessing physical capability in older adults), compared to those with higher 25(OH)D levels. In addition, calf muscle cross-sectional area loss was also greater. Vitamin D deficient patients without PVD had faster declines in walking velocity. There were no associations between 25(OH)D levels and mortality, although the total number of deaths was relatively small (*n* = 93) [67].

## 10. Vitamin D and Overall Mortality

Most observational studies have associated a low vitamin D status with an increased risk of death [68, 69]. A number of RCTs have investigated the effect of pharmacological supplementation of vitamin D and subsequent risk of overall mortality. In a meta-analysis of 51 RCTs vitamin D supplementation did not have a significant impact on mortality (RR, 0.96%; 95% CI, 0.93–1.00; *P* = 0.08) [70]. There is evidence that supplementation with vitamin D combined with calcium but not vitamin D alone may improve overall mortality. An individual patient meta-analysis of eight major vitamin D trials including 70,528 patients (86.8% females with median age of 70) found vitamin D alone did not affect mortality but when supplemented with calcium this reduced mortality by 7% (HR, 0.91; 95% CI, 0.84–0.98) [71]. The authors calculated that the number needed to treat with vitamin D plus calcium was 151 for 3 years to prevent 1 death. Unfortunately, baseline vitamin D levels were not performed on these patients. Clearly, whether vitamin D supplementation provides a mortality benefit with or without calcium supplements is a very important issue for future research.

A meta-analysis of 50 RCTs where vitamin D was administered for median of 2 years found vitamin D3 to significantly decrease mortality (RR, 0.94; 95% CI, 0.91–0.98; 32 trials), whilst vitamin D2, alfacalcidol, and calcitriol had no statistical effect on mortality [72]. Furthermore, trials looking into baseline vitamin D status using cutoffs of above and below 50 nmol/L, dosing above or below 800 IU/d, daily or intermittent dose frequency, and the addition or absence of calcium all had no significant effects on mortality, though this could be due to type II errors. When vitamin D3 was combined with calcium, there was a significant increase in nephrolithiasis (RR, 1.17; 95% CI, 1.02–1.34; *P* = 0.02). Furthermore, alfacalcidol and calcitriol increased the risk of hypercalcaemia (RR, 3.18; 95% CI, 1.17–8.68; *P* = 0.02). The authors concluded that vitamin D3 seemed to decrease mortality in predominantly elderly women who are not dependent on help or living in institutional care.

Most of the data conducted are on vitamin D in primary prevention trials; thus it is difficult to draw any conclusions with regard to vitamin D in secondary prevention because of the lack of evidence currently available. When looking at trial data in primary prevention, there is risk for attrition bias originating from substantial dropout of participants and also outcome reporting bias due to investigators not reporting mortality. Thus, the available information of vitamin D and mortality is inconclusive and therefore further placebo controlled RCTs are needed.

## 11. Vitamin D Supplementation Recommendations

Oral vitamin D3 taken at physiological doses is effective in raising blood 25(OH)D levels and can be taken daily, weekly, or monthly as it produces comparable increases in 25(OH)D levels [73]. None of the major cardiology societies have made recommendations for the optimum supplementation for cardiovascular disease. Based on the positive effect for musculoskeletal health, the Institute of Medicine (IOM) in 2011 recommended minimum vitamin D levels of 50 nmol/L (≥20 ng/mL) [74] (Table 3). Therefore, their guidance for daily vitamin D intake in the general population is 600 IU/d for individuals up to 70 years old and 800 IU/d in the older individuals which corresponded to the recommended daily allowance (covering requirements of ≥97.5% of the general population). The US Endocrine Society suggests 1500–2000 IU/d of vitamin D is needed for adults to maintain 25(OH)D above the optimal level of 75 nmol/L [75]. In the UK, only at risk groups are recommended for supplementation, at 10 micrograms/day (400 IU/d), although the categories included are very broad [76]. With regard to safety, both the official statements of the IOM and the European Food Safety Authority declare that daily intakes up to 4000 IU are safe [74, 77]. Only in exceedingly rare cases, exposure to extraordinarily high pharmacological doses of vitamin D has caused sequelae [78]. Despite this, we should be cautious with high-dose vitamin D supplementation as there is some suggestion of a J-shaped association between 25(OH)D levels and mortality [79].

## 12. Conclusions

There are weak signals of benefit of vitamin D supplementation in heart failure, whilst studies have so far shown vitamin D to be ineffective as an agent in lowering blood pressure and thus it should not be used as an antihypertensive agent. In CAD, AF, PVD, and stroke, although low vitamin D levels are associated with disease occurrence, supplementation has not yet been shown to be of benefit. Whether vitamin D is a causal factor or convenient biomarker is not yet established and maybe supplementation in cohorts of older adults may be too late. Modest reductions in all-cause mortality by vitamin D3 were demonstrated by meta-analysis [71], but the number needed for benefit was around 150 and, due to attrition bias, the reliability of the estimate was low.

On the other hand, vitamin D deficiency is very prevalent and modest improvements might make substantial health gains, with a low risk of adverse outcome. On the strength of current evidence, supplementation for optimum cardiovascular outcomes alone does not seem to be a justifiable position, but, for overall health, following IOM [74] and National Institute for Health and Care (NICE) guidance [80] is the best effort to answer an underinvestigated question. A number of areas, particularly heart failure, warrant controlled trials look for robust evidence for a potentially cheap, low risk, and effective treatment option.

## Figures and Tables

**Table 1:** Proposed classification of vitamin D status.

Vitamin D status	nmol/L	ng/mL
Toxicity	>375	>150
Optimal	100–150	40–60
Sufficient	75–99	30–39
Insufficient	50–74	20–29
Deficient	<50	<20

**Table 2:** Key studies in vitamin D and cardiovascular disease.

Disease	Study	Design	Number of participants	Outcome
Hypertension	Scragg et al. (2007) [22]	Cross-sectional, observational	12,644	Vitamin D status is inversely associated with BP.
	Beveridge et al. (2015) [26]	Meta-analysis of 46 RCTs	4541	No effect of Vitamin D supplementation on systolic or diastolic BP.
	Forman et al. (2013) [27]	RCT	283	Dose dependent reduction in systolic BP.
	Dong et al. (2010) [28]	RCT	49	Reduction in arterial stiffness in young African-Caribbean subjects.

Heart failure	Zittermann et al. (2003) [34]	Observational	88	Chronic heart failure is more common in groups with low vitamin D.
	van Ballegooijen et al. (2013) [35]	Cross-Sectional, observational	969	Higher PTH was associated with greater left ventricular mass and lower systolic function.
	Kestenbaum et al. (2011) [36]	Observational	2,312	Vitamin D deficiency is associated with MI, mortality, and excess PTH with heart failure.
	Saleh et al. (2003) [37]	Observational	2,700	PTH is associated with left ventricular mass.
	Shedeed (2012) [38]	RCT	80	Supplementation improved cardiac function and decreased PTH and inflammatory markers.
	Schleithoff et al. (2006) [39]	RCT	93	Supplementation reduced inflammatory mediators and parathyroid hormone.
	Gotsman et al. (2012) [40]	Observational	49,834	Vitamin D deficiency highly prevalent and predictive of reduced survival.

Atrial fibrillation	Frost et al. (2002) [42]	Retrospective, observational	32,992	Seasonal variation in AF incidence, showing a winter peak and summer trough.
	Murphy et al. (2004) [43]	Retrospective, observational	68,045	Winter peak of hospitalizations with AF, with higher winter mortality.
	Reinstra et al. (2011) [44]	Retrospective, observational	2,930	Vitamin D is not associated with AF.
	Qayyum et al. (2012) [45]	Observational	258	Vitamin D is not associated with AF, stroke, or MI.
	Demir et al. (2014) [46]	Case-control	298	Vitamin D is associated with nonvalvular AF.
	Chen et al. (2014) [47]	Case-control	162	Association with AF and vitamin D deficiency are shown.
	Ozcan et al. (2015) [48]	Case-control	227	Vitamin D deficiency was associated with new-onset AF.

Coronary artery disease	Thomas et al. (2012) [50]	Cohort	1,801	Reduced all-cause and cardiovascular mortality and heart failure but not MI.
	Wang et al. (2008) [51]	Cohort	1,739	Higher risk of cardiovascular events with lower vitamin D levels.
	Oz et al. (2013) [52]	Cross-sectional, observational	222	Vitamin D deficiency associated with slow coronary flow, endothelial dysfunction, and subclinical atherosclerosis.
	Liew et al. (2015) [53]	Observational	375	Low vitamin D associated with worse angiographic CAD, nonarterial stiffness, or PVD.
	Ford et al. (2014) [54]	RCT	5,292	Vitamin D is protective against heart failure but not MI and stroke.

Stroke	Witham et al. (2012) [59]	RCT	58	High dose of vitamin D improved endothelial function in stroke patients with controlled BP.
	Hsia et al. (2007) [60]	RCT	36,282	No observed changes in coronary or stroke risk.
	Avenell et al. (2012) [61]	RCT	5,292	No effect on mortality, vascular disease, cancer mortality, or cancer incidence.
	Trivedi et al. (2003) [62]	RCT	2,686	Fractures may be reduced by supplementation but no change in stroke risk.

Peripheral vascular disease	Anderson et al. (2010) [63]	Retrospective, observational	41,504	Vitamin D deficiency associated with diabetes, hyperlipidemia, HTN, CAD, PVD, MI, and stroke.
	Melamed et al. (2008) [64]	Retrospective, observational	4,839	Low vitamin D is associated with higher PVD.
	Fahrleitner et al. (2002) [65]	Cross-sectional, observational	327	PVD is associated with vitamin D deficiency, secondary hyperparathyroidism, and osteomalacia.
	McDermott et al. (2014) [67]	Observational	658	Lower vitamin D levels associated with faster decline in walking in those with PVD.

Mortality	Zittermann et al. (2012) [68]	Meta-analysis of prospective cohorts	62,548	Nonlinear decrease in mortality for increased vitamin D levels, plateauing around 87.5 nmol/L.
	Johansson et al. (2012) [69]	Observational	2,878	In elderly men, low vitamin D levels are associated with increased mortality.
	Elamin et al. (2011) [70]	Meta-analysis of 51 RCTs	51 RCTs	No evidence of significant reduction in mortality or cardiovascular risk.
	Rejnmark et al. (2012) [71]	Meta-analysis of 8 major trials	70,528	Vitamin D with calcium reduced overall mortality.
	Bjelakovic et al. (2014) [72]	Meta-analysis of 56 RCTs	95,286	Vitamin D3 seemed to decrease mortality in elderly people.

BP, blood pressure; PTH, parathyroid hormone; AF, atrial fibrillation; MI, myocardial infarction; CAD, coronary artery disease; PVD, peripheral vascular disease; HTN, hypertension; RCT, randomised controlled trial.

**Table 3:** Recommended daily vitamin D supplementation for adults.

Body	Recommendation	Daily dose
Institute of Medicine [74]	Supplementation for age groups	
	(i) Individuals up to 70 years old	600 IU
	(ii) Older individuals (>70 years old)	800 IU

National Institute for Health and Care Excellence [80] and Public Health England [76]	Supplementation for at risk groups (i) Pregnant and breastfeeding women, particularly young women. (ii) People over 65. (iii) People who have low or no exposure to the sun, for example, those who cover their skin for cultural reasons and who are housebound or confined indoors for long periods. (iv) People with darker skin, for example, people of African, African-Caribbean, or South Asian family origin.	10 micrograms (400 IU)

IU, international units.
